# Photochemical Finish: Assessing the Genomic Impact of Secondary Air Pollutants

**DOI:** 10.1289/ehp.119-a488b

**Published:** 2011-11-01

**Authors:** Bob Weinhold

**Affiliations:** Bob Weinhold, MA, has covered environmental health issues for numerous outlets since 1996. He is a member of the Society of Environmental Journalists.

Most studies and regulations of air pollutants address individual substances in their unaltered primary form. But people typically are exposed simultaneously to multiple pollutants, and primary pollutants can be photochemically transformed in the atmosphere into secondary, tertiary, and additional generations of substances. Scientific limitations have hampered study of these real-world conditions and impaired our understanding of exactly how air pollutants affect health. Findings by a team of University of North Carolina researchers provide insight into the biological effects of air pollution and suggest that current air pollution regulations and mitigation approaches should consider products of atmospheric reactions, which have received scant attention in the past [*EHP* 119(11):1583–1589; Rager et al.].

The researchers used a toxicogenomics approach to evaluate the impacts of primary and secondary pollutants on human epithelial lung cells. They found that 4 hours of exposure to a mixture composed largely of primary pollutants (nitric oxide, nitrogen dioxide, and 55 hydrocarbons) at environmentally relevant concentrations changed the expression of 19 genes, compared with 709 altered genes following similar exposure to a mixture of primary and secondary pollutants (including ozone, formaldehyde, and peroxyacetyl nitrate). They also found that release of the enzyme lactate dehydrogenase, a marker of cell injury and death, was 9 times higher after exposure to the secondary pollutants compared with the primary pollutants.

The authors identified molecular networks related to sets of genes expressed in response to exposure. One network was identified for genes affected by the primary pollutant mixture, which included proteins relevant to cancer biology. Twenty-five networks were identified for genes affected by the secondary pollutants, including those relevant to biologic processes associated with cancer; cellular movement, growth, and proliferation; tissue development; and cardiovascular disease. The authors also identified transcription factors that might regulate responses to the pollutant exposures. Both primary and secondary pollutants affected hepatocyte nuclear factor 4α signaling, which plays a role in the function of organs such as the kidney, liver, and intestine.

The authors acknowledge this study offers only a glimpse of what’s occurring in real-world conditions. Future studies will need to consider long-term exposures, defense mechanisms in living beings, specific health impacts linked with altered gene expression, many other primary pollutants, and myriad combinations of primary, secondary, and subsequent generations of pollutants. In addition, research is needed to pin down the roles of individual substances within any given mixture. But these initial results provide insights into what is missing in current knowledge and should spark more in-depth investigations.

